# Effects of reduced follicle-stimulating hormone dosage before human chorionic gonadotropin trigger on in vitro fertilization outcomes

**DOI:** 10.1186/s12884-023-05943-5

**Published:** 2023-08-25

**Authors:** Zhanhui Ou, Jing Du, Nengqing Liu, Jieliang Li, Xiufeng Lin

**Affiliations:** 1 Reproductive Medicine Center, Boai Hospital of Zhongshan Affiliated to Southern Medical University, 6 Chenggui Road, East District, Zhongshan, 528400 Guangdong China; 2 The Second School of Clinical Medicine, Southern Medical University, Guangzhou, 510515 Guangdong China

**Keywords:** Ovarian stimulation, hCG trigger, Fresh embryo transfer, Progesterone, In vitro fertilization

## Abstract

**Objective:**

To determine whether a reduced dose of follicle-stimulating hormone (FSH) before human chorionic gonadotropin (hCG) trigger during ovarian stimulation can affect in vitro fertilization (IVF) outcomes.

**Methods:**

This study included 347 patients with a normal ovarian response who received a reduced dose of FSH before hCG trigger for 2–3 days (Group A) and 671 patients who did not receive a reduced dose (Group B) from a university-affiliated IVF center between January 2021 and December 2022. The primary endpoint was estrogen (E2) and progesterone (P) levels on the day of hCG trigger, fresh embryo transfer cycles, laboratory outcomes, and clinical outcomes between the two groups.

**Results:**

On the day of hCG trigger, Group A had significantly lower E2 and P levels than those in Group B (3454.95 ± 1708.14 pg/mL versus 3798.70 ± 1774.26 pg/mL, p = 0.003; and 1.23 ± 0.53 ng/mL versus 1.37 ± 0.59 ng/mL, p < 0.001, respectively). The proportion of patients with P levels ≥ 1.5 ng/mL was 22.48% in Group A compared to 34.58% in Group B (p < 0.001), while the proportion of patients with E2 ≥ 5000 pg/mL was 15.27% in Group A compared to 25.93% in Group B (p < 0.001). The fresh embryo-transfer cycle rate in Group A was higher than that in group B (54.47% and 32.64%, respectively; p < 0.001). Despite the reduction in FSH dosage, there were no significant differences between groups regarding the number of oocytes retrieved, total number of mature oocytes, normal fertilization rate, cleavage rate, Day 3 top-quality rate, implantation rate, pregnancy rate per cycle, and early pregnancy loss rate.

**Conclusion:**

While a reduced dose of FSH prior to hCG trigger during ovarian stimulation did not significantly affect IVF outcomes, it was associated with lower E2 and P levels, resulting in fewer cycles with E2 ≥ 5000 pg/mL and P ≥ 1.5 ng/mL on the day of the hCG trigger.

## Introduction

Frozen embryo transfer (FET) is a widely used method with several advantages. It has a lower risk of ovarian hyperstimulation syndrome (OHSS) [1, 2], provides a favorable intrauterine environment for embryo implantation and placentation by avoiding supra-physiologic hormonal levels after ovarian stimulation [3, 4], and can improve pregnancy and live birth rates compared to fresh embryo transfer [5, 6]. However, there are also complications, including an increased risk of macrosomia, large for gestational age, and preeclampsia [6–9]. Moreover, fresh embryo transfers may shorten the embryo transfer time and is more cost-effective; therefore, it is the most common and recommended practice in most in vitro fertilization (IVF) units in China. Increased serum progesterone (P) levels may occur in the late follicular phase during ovarian stimulation with *gonadotropin-releasing hormone (*GnRH) agonist and antagonist protocols in approximately 35% and 38% of women, respectively [10, 11]. This may be detrimental to successful IVF implantation as it advances endometrial histology and impairs endometrial receptivity [11–14]. Bosch et al. (2003) demonstrated a decrease in ongoing pregnancy rates with serum P levels ≥ 1.5 ng/mL on the day of human chorionic gonadotropin (hCG) administration. Moreover, distinct differences in endometrial gene expression have been found in patients with increased serum P levels on hCG administration [13, 15]. Additionally, high levels of estrogen (E2) have been associated with OHSS [16]. Kyrou et al. (2012) previously demonstrated that patients with high E2 level also have high P levels [17]; hence, P levels ≥ 1.5 ng/mL or E2 levels above 5000 pg/mL, indicate that embryos should be frozen after oocyte retrieval.

Underlying mechanisms for increased E2 and P levels during the follicular phase and their roles in reducing pregnancy rates are unclear. Some studies have shown that synthesis of P through preovulatory follicles is stimulated by the follicle-stimulating hormone (FSH) and luteinizing hormone (LH) [18]. To prevent increased E2 and P level, methods, including a different gonadotropin [19], double GnRH antagonist dose (0.25 mg/12 h) the day before hCG administration [20], and step-down FSH dosage during ovarian stimulation have been suggested [21]. Ozgur et al. (2017) treated the granulosa cells in vitro with recombinant FSH (r-FSH); FSH significantly increased the P and E2 protein expressions [18]. Additionally, patients with more follicles and oocytes have been found to possess higher P levels [22, 23]. Thus, gonadotropin stimulation or the degree of ovarian stimulation may have significantly impacted the P levels before hCG trigger. However, Barbara et al. revealed that a step-down approach of daily 12.5 international units (IU) of r-FSH did not significantly reduced the P levels on the day of hCG trigger, but was associated with the total gonadotropin-stimulation dosage [21].

To our knowledge, there are few studies on the step-down of FSH dosage during ovarian stimulation in patients showing a normal ovarian response, except for those with OHSS [24, 25]. This study aimed to compare the IVF outcomes, E2 and P levels on hCG trigger between patients showing a normal ovarian response and those administered a reduced FSH dose (50–100 IU) before hCG trigger, and patients without a reduced dose.

## Materials and methods

### Study design and participants

This retrospective cohort study analyzed the medical data of patients who received a reduced dose of FSH (50–100 IU) before hCG trigger for 2–3 days against those who did not receive a reduced dose between January 2021 and December 2022 in our center. The exclusion criteria were as follows: [1] patients with pre-implantation genetic testing cycles; [2] age ≥ 35 years; [3] anti-Müllerian hormone (AMH) ≥ 4.0 µg/L or ≤ 1.1 µg/L; [4] antral follicle counts (AFC) ≥ 15 or < 5, basal FSH ≥ 10 U/L; [5] body mass index (BMI) > 30; [6] congenital or acquired uterine malformations; [7] endometriosis; [8] abnormal results on parental karyotyping; and [9] medical conditions that contraindicated assisted reproductive technology or pregnancy. Only healthy patients with a normal ovarian response were included in this study. Our final cohort included 347 patients showing a normal ovarian response with a reduced dose of FSH before hCG trigger for 2–3 days (Group A) and 671 patients without a reduced dose of FSH (Group B).

### Ethical approval

This study was approved by the Ethics Committee of Zhongshan Boai Hospital (KY-2023-04-02). Informed consent was obtained from the enrolled patients.

### Patient treatment

Standard controlled ovarian stimulation protocols were utilized. The patients were briefly treated with either the GnRH agonist or GnRH antagonist protocol for ovulation induction. Ovarian stimulation was performed using r-FSH (Gonal-F, Merck-Serono, Geneva, Switzerland) in combination with a GnRH agonist (Triptorelin Acetate, Ipsen Pharma Biotech, France) or a GnRH antagonist (Cetrorelix Acetate, Merck-Serono, Geneva, Switzerland). Initial doses were based on previous recommendations by the European Society of Human Reproduction and Embryology (ESHRE) and regulated according to AFC, age, BMI, AMH, and basal FSH levels [26]. When the follicles reached a mean diameter of approximately 15 mm, a reduced dose of FSH (50 IU–100 IU) was given before the hCG trigger for 2–3 days in Group A. Group B did not receive a reduced dose. Additionally, hCG or GnRH agonist was administered when at least three leading follicles reached a mean diameter of ≥ 18 mm. Transvaginal oocyte retrieval was scheduled about 36 h later.

### Embryo transfer

Fresh embryos were transferred on Day 3 or 5 except in case of: [1] serum P was > 1.5 ng/mL or E2 ≥ 5000 pg/mL on the trigger day; [2] endometrial thickness < 7 mm on the trigger day; and [3] endometrial abnormalities. P was administered after oocyte retrieval for daily fresh embryo transfer cycles. A maximum of two embryos were transferred on Day 3 or 5 after oocyte retrieval. Embryos were transferred under the guidance of a trans-abdominal ultrasound. Luteal support was given for approximately 10 weeks after oocyte retrieval.

### Outcome measures

Hormone levels, including E2 and P, on the trigger day of hCGand the proportion of patients with P levels ≥ 1.5 ng/mL and thosewith E2 ≥ 5000 pg/mL were recorded. Laboratory outcomes included the number of oocytes retrieved, number of metaphase II (MII) oocytes, normal fertilization rate, cleavage rate, and the good quality embryo rate on Day 3. The clinical outcomes included the number of fresh embryo transfer cycles, implantation rate, clinical pregnancy rate per cycle transfer, and miscarriage rate per pregnancy.

### Statistical analyses

Data were presented as the means ± standard deviations. When the data distribution passed the normality test, the data of the two experimental groups were compared using the 2-tailed Student t-test (age, BMI, AFC, duration of stimulation, total gonadotrophin dose, AMH, basal FSH, E2, P, number of abortions, endometrium the day of the hCG trigger [Table 1]; P and E2 on the day of hCG trigger, number of oocytes retrieved, number of MII oocytes [Table 2]; embryo transfer [Table 3]. The chi-square test was used to analyze the fertilization rate, clinical pregnancy rate, and other data (infertility type, stimulation protocols [Table 1]; P ≥ 1.5 ng/mL, E2 ≥ 5000 pg/mL, fertilization rate, cleavage rate, Day 3 top-quality rate [Table 2]; fresh ET cycles, implantation rate, pregnancy rate per cycle, early pregnancy loss [Table 3]. P < 0.05 was considered statistically significant.

**Table 1:** Demographic information of the patients from the two groups

Variable	Group A (347)	Group B (671)	P-value
Age (years)	30.76 ± 2.75	30.45 ± 2.58	0.064
BMI (kg/m^2^)	21.84 ± 3.62	21.50 ± 3.38	0.136
AFC	10.50 ± 2.57	10.65 ± 2.47	0.352
Duration of stimulation (days)	10.79 ± 1.45	10.68 ± 1.44	0.230
Total gonadotrophin dose	2619.95 ± 601.53	2685.55 ± 683.73	0.116
AMH	2.58 ± 0.75	2.65 ± 0.73	0.119
Basal FSH	7.45 ± 1.39	7.39 ± 1.36	0.550
Basal E2	40.16 ± 29.17	40.41 ± 31.72	0.900
Basal P	0.58 ± 0.50	0.64 ± 0.92	0.276
Number of abortions	0.45 ± 0.82	0.44 ± 0.74	0.936
Infertility type			0.981
Primary infertility	45.53% (158/347)	45.45% (305/671)	
Secondary infertility	54.47% (189/347)	54.55% (366/671)	
Stimulation protocols			0.495
Antagonist protocol	25.94% (90/347)	23.99% (161/671)	
Agonist protocol	74.06% (257/347)	76.01% (510/671)	
Endometrium the day of the hCG trigger	10.56 ± 2.19	10.78 ± 2.24	0.129

Abbreviations: hCG, human chorionic gonadotropin; E2, estrogen; P, progesterone; AMH, anti-Müllerian hormone: BMI, body mass index; FSH, follicle-stimulating hormone. AFC: Antral Follicle Counting

**Table 2:** Laboratory outcomes of the patients from the two groups

Outcome	Group A (347)		Group B (671)		P-value	
P on the day of hCG trigger	1.23 ± 0.53		1.37 ± 0.59		0.000	
E2 on the day of hCG trigger	3454.95 ± 1708.14		3798.70 ± 1774.26		0.003	
P ≥ 1.5 ng/mL	22.48% (78/347)		34.58% (232/671)		0.000	
E2 ≥ 5000 pg/mL	15.27% (53/347)		25.93% (174/671)		0.000	
No. of oocytes retrieved	10.59 ± 4.83		10.64 ± 4.62		0.872	
No. of MII oocytes	9.40 ± 4.57		9.56 ± 4.46		0.586	
Fertilization rate, n (%)	IVF	ICSI	IVF	ICSI	IVF P	ICSI P
	64.49% (1712/2655)	80.30% (685/853)	66.09% (3341/5055)	81.31% (1470/1808)	0.157	0.539
Cleavage rate, n (%)	98.41% (2359/2397)		98.82% (4754/4811)		0.160	
Day 3 top-quality rate, n (%)	62.02% (1464/2359)		63.04% (2997/4754)		0.420	

Abbreviations: hCG, human chorionic gonadotropin; E2, estrogen; P, progesterone; IVF, in vitro fertilization; ICSI, intracytoplasmic sperm injection; MII: metaphase II.

**Table 3:** Clinical outcomes of the patients from the two groups Abbreviation: ET, embryo transfer; SD, standard deviation

Outcome	Group A (347)	Group B (671)	P
Fresh ET cycles	54.47% (189/347)	32.64% (219/671)	0.000
ET (mean ± SD)	1.07 ± 0.25	1.09 ± 0.28	0.502
Implantation rate, n (%)	43.56% (88/202)	37.82% (90/238)	0.221
Pregnancy rate per cycle, n (%)	44.44% (84/189)	38.36% (84/219)	0.213
Early pregnancy loss, n (%)	8.33% (7/84)	11.90% (10/84)	0.443

## Results

### Demographics outcomes

No significant differences were found regarding the mean age of the patients, BMI, AFC, AMH, basal FSH levels, basal P levels, basal E2 levels, rate of primary infertility; stimulation protocols, number of abortions, and thickness of the endometrium on the day of the hCG trigger between the groups (All *P* > 0.05) (Table 1). Additionally, no significant difference was reported in the total duration of FSH stimulation (2619.95 ± 601.53 versus 2685.55 ± 683.73 IU, *P* = 0.116) and average stimulation days (10.79 ± 1.45 versus 10.68 ± 1.44, *P* = 0.230) between Group A and Group B (Table 1).

### Hormone outcomes

On the day of the hCG trigger, patients in Group A had significantly lower E2 levels compared to those in Group B (3454.95 ± 1708.14 versus 3798.70 ± 1774.26 pg/mL, *P* = 0.003). A similar result was observed for the P levels on the day of the hCG trigger between the two groups (1.23 ± 0.53 versus 1.37 ± 0.59, *P* < 0.001). The proportion of patients with P levels ≥ 1.5 ng/mL was 22.48% (78/347) in Group A versus 34.58% (232/671) in Group B (*P* < 0.001), while the proportion of patients with E2 ≥ 5000 pg/mL was 15.27% (53/347) in Group A versus 25.93% (174/671) in Group B (*P* < 0.001) (Table 2).

### Laboratory outcomes

Day 3 top-quality rate was 62.02% (1464/2359) for Group A versus 63.04% (2997/4754) for Group B (*P* = 0.420). No significant differences were observed regarding the number of oocytes retrieved (10.59 ± 4.83 versus 10.64 ± 4.62, *P* = 0.872), number of MII oocytes (9.40 ± 4.57 versus 9.56 ± 4.46, *P* = 0.586), normal fertilization rate of IVF (64.49% [1712/2655] versus 66.09% [3341/5055], *P* = 0.157), normal fertilization rate of ICSI (80.30% [685/853] versus 81.31% [1470/1808], *P* = 0.539), and cleavage rate (98.41% [2359/2397] versus 98.82% [4754/4811], *P* = 0.160) between the two groups (Table 2).

### Clinical outcomes

Fresh embryo transfer cycles were higher in Group A (54.47%, 189/347 patients), than in Group B (32.64%, 219/671 patients) (*P* < 0.001). However, no significant differences were observed between the two groups regarding the number of embryos transferred (1.07 ± 0.25 versus 1.09 ± 0.28, *P* = 0.502), implantation rate (43.56% [88/202] versus 37.82% [90/238], *P* = 0.221), pregnancy rate per cycle rate (44.44% [84/189] versus 38.36% [84/219], *P* = 0.213), and early pregnancy loss rate (8.33% [7/84] versus 11.90% [10/84], *P* = 0.443) (Table 3).

## Discussion

In our study, we retrospectively analyzed and compared the data of patients showing normal ovarian response who received a reduced dose of FSH before hCG trigger for 2–3 days (Group A) with those of patients who did not receive a reduced dose during ovarian stimulation (Group B). Our results demonstrated that on hCG administration, Group A had significantly lower E2 and P levels than Group B. Numerous studies have been conducted regarding the relationship between FSH dose and E2 and P levels. For instance, Ozgur et al. (2017) treated granulosa cells in vitro with r-FSH, and found that r-FSH significantly increased the protein expression of P and E2 [18]. Additionally, an in vivo study revealed that FSH dose positively correlates with P concentrations [27].

Previous studies have shown that E2 ≥ 5000 pg/mL is associated with a high risk of OHSS, and P ≥ 1.5 ng/mL may affect pregnancy rates [10]. To further study the effect of a reduced dose of FSH on fresh embryo transfer, the proportion of patients with E2 ≥ 5000 pg/mL and P ≥ 1.5 ng/mL was compared between Groups A and B. We found that the proportion of patients with E2 ≥ 5000 pg/mL and P ≥ 1.5 ng/mL was lower in Group A than in Group B. These results suggest that patients showing a normal ovarian response treated with a reduced dose of FSH before hCG trigger may achieve lower levels of E2 and P and thus increase the possibility of fresh embryo transfer. We think that this method is similar to the “Coasting” method, which is a well-known strategy to decrease severity of OHSS [28, 29]. Coasting involves the withdrawal of exogenous gonadotrophins once an optimal follicular size is achieved and the withholding of HCG until the serum estradiol concentration decreases to a ‘safe’ concentration without effecting the live birth and clinical pregnancy rate [28, 29]. Further, some investigators recommend that patients started Coasting when more than 15 to 20 follicles of > 15 mm are detected and the serum E2 levels rise to 4500 pg/mL [30]. This may be more complicated than our method for use in clinic, which only focuses on reducing the dose of FSH before hCG trigger without withholding HCG until the serum estradiol concentration decreases.

Laboratory and clinical outcomes revealed no significant differences between the two groups in our study. These results were concordant with those of a previous study in which a reduced dose of gonadotropin or GnRH antagonist administered twice a day before hCG trigger combined with a step-down protocol did not affect oocyte retrieval, fertilization rate, implantation rate, or pregnancy rate [20]. There are two potential reasons for this result. First, daily injections of FSH can lead to an accumulation of exogenous gonadotropins in the patient’s serum. r-FSH (Gonal-F, Merck-Serono, Geneva, Switzerland) has a half-life of approximately 42.58 h after a single injection at a dose of 225 IU [31]. This hypothesis was confirmed in another study that utilized a stepwise reduction in the daily dose of FSH (12.5 IU) over a median time period of 3 days. Serum FSH levels remained constant until the day of the trigger, and no difference was found between FSH serum levels on the day of hCG trigger in the stepwise-reduction and control groups [21]. Second, in the early antral follicle stage, the granulosa cells are sensitive to FSH stimulation. Induction of LH receptor expression in the granulosa cells is mainly regulated by FSH, and as the follicle matures into the preovulatory stage, induction of LH receptor expression occurs more effectively than that of FSH receptor expression [32]. When the follicle is over 14 mm in size, the numbers of FSH and LH receptors change, with a decline in the number of FSH receptors and a parallel increase in the number of LH receptors. This mechanism may explain why the reduced dose of FSH had no impact on further follicle development and oocyte yield.

In our study, only patients showing a normal ovarian response were included; a poor or high ovarian response may affect the E2 levels, P levels, laboratory outcomes, and clinical outcomes [33–37]. Moreover, the stimulation protocols and dose of treatment may vary greatly between patients with poor or high ovarian response. Further, patients with a reduced dose of FSH < 50 IU before hCG trigger for < 2 days were also excluded, as the low dose and short duration of FSH reduction may not be sufficient to produce meaningful results.

There are some limitations to this study. First, because this was a retrospective study, selection bias cannot be excluded. Thus, randomized control trials are required to eliminate this effect. Second, this study possessed a relatively small sample size; therefore, to better understand the relationship between a reduced dose of FSH before hCG trigger during ovarian stimulation and IVF outcomes, studies with larger sample sizes are required. Third, the starting doses of gonadotropin stimulation were not consistent between the two groups, because these doses were based on AFC, age, BMI, and AMH of the patients.

In conclusion, while a reduced dose of FSH before hCG trigger during ovarian stimulation did not affect IVF outcomes, it may lower E2 and P levels, resulting in fewer cycles with E2 ≥ 5000 pg/mL and P ≥ 1.5 ng/mL on the day of hCG trigger.

## Data Availability

Data were obtained from the referenced publications. Further information is available by contacting Dr. Ou at zhanhui-ou@hotmail.com.

## Abbreviations

FSH: Follicle-stimulating hormones
hCG: Human chorionic gonadotropin
IVF: In vitro fertilization
E2: Estrogen; P:progesterone
FET: Frozen embryo transfer
OHSS: Ovarian hyperstimulation syndrome
GnRH: Gonadotropin-releasing hormone
LH: Luteinizing hormone
r-FSH: Recombinant FSH
IU: International units
PGT: Pre-implantation genetic testing
AMH: Anti-Müllerian hormone
AFC: Antral follicle counts
BMI: Body mass index
